# Beyond counting induced abortions, miscarriages and stillbirths to understanding their risk factors: analysis of the 2017 Ghana maternal health survey

**DOI:** 10.1186/s12884-021-03633-8

**Published:** 2021-02-16

**Authors:** Bright Opoku Ahinkorah, Abdul-Aziz Seidu, Edward Kwabena Ameyaw, Eugene Budu, Freda Bonsu, Bupe Mwamba

**Affiliations:** 1 School of Public Health, Faculty of Health, University of Technology Sydney, Sydney, Australia; 2 Department of Population and Health, College of Humanities and Legal Studies, University of Cape Coast, PMB University Private Mail Bag, Cape Coast, Ghana; 3 College of Public Health, Medical and Veterinary Sciences, James Cook University, 4811 Townsville, Queensland Australia; 4 Asutifi South District Health Directorate, Hwidiem, Ghana; 5 Centre for Midwifery, Family and Child Health, Faculty of Health, University of Technology Sydney, Sydney, Australia

**Keywords:** Induced abortion, Stillbirth, Miscarriage, Pregnancy outcomes, Women, Maternal health, Ghana, Maternal Health survey

## Abstract

**Background:**

Inasmuch as induced abortions, miscarriages and stillbirths constitute common adverse pregnancy outcomes contributing to poor maternal health, there is paucity of literature about these in Ghana. We investigated the factors associated with induced abortions, miscarriages and stillbirths in Ghana.

**Methods:**

Data derived from the 2017 Ghana Maternal Health Survey was used in this study. Women aged 15–49 constituted the target for the study. This study examined the relationship between socio-demographic characteristics and induced abortions, stillbirths and miscarriages. Subsequently, multivariable binary logistic regression models were fitted to investigate the factors associated with induced abortions, stillbirths and miscarriages at 95 % confidence interval (CI).

**Results:**

The prevalence of miscarriages, induced abortions and stillbirths in Ghana in 2017 were 10.8 %, 10.4 % and 2 % respectively. Induced abortions (12.9 %) and miscarriages (11.1 %) were found to be higher among urban residents whiles rural residents had more of stillbirths (2.1 %). Compared to women aged 15–24, those in all age categories had lower odds of experiencing induced abortions, with the lowest odds occurring among women aged 35–49 (AOR = 0.26, 95 % CI = 0.21-32). Conversely, women of all age categories had higher odds of experiencing miscarriages compared to those aged 15–24 with the highest odds among those aged 25–34 (AOR = 1.62, 95 % CI = 1.39–1.89). Women with at least primary education were more likely to experience miscarriages than those with no formal education, with those with higher level of education having the highest odds (AOR = 1.42, 95 % CI = 1.13–1.78). While the likelihood of induced abortions was lower among Muslims, compared to Christians (AOR = 0.65, 95 % CI = 0.52–0.82), the odds of miscarriages were higher among Muslims, compared to Christians (AOR = 1.31, 95 % CI = 1.13–1.52). Women with parity 1 or more were less likely to experience induced abortions, miscarriages and stillbirths compared to those with parity 0.

**Conclusions:**

Our study indicates that efforts to limit induced abortions, miscarriages and stillbirths in Ghana need to focus on the disparities in socio-demographic characteristics of women. Synergy between government health institutions and the private sector cannot be left out if much success can be achieved in efforts to subside the current prevalence of induced abortions, stillbirths and miscarriages confronting the country.

## Background

Globally, induced abortions, stillbirths and miscarriages are among the major maternal and child health concerns [1]. Stillbirths and miscarriages, for instance, are common adverse pregnancy outcomes that contribute significantly to poor maternal health [1, 2]. Induced abortion is the intentional termination of pregnancy. This can be safe or unsafe [3]. The unsafe termination involves terminating a pregnancy by a person that lacks the necessary skills or in an environment, not in conformity with minimal medical standards, or both [4]. The WHO [5] defines stillbirth as the death of a foetus in the uterus before birth, at or after 28-weeks gestational age. The five main causes of stillbirths are childbirth complications, maternal infections in pregnancy, maternal disorders (especially pre-eclampsia and diabetes), fetal growth restriction and congenital abnormalities [5–7]. Miscarriage on the other hand, also known as spontaneous abortion, refers to an untriggered foetal loss before 22 weeks of gestation [8]. The reported causes of miscarriage include genetic factors, uterine anatomical defects, infection, endocrine, and immunological factors. Maternal age and the number of prior miscarriages/spontaneous abortions are also associated with recurrent miscarriages [9].

Globally, among 136 million babies born every year, approximately 4 million are stillborn [10]. The estimated stillbirth rate for high-income countries ranges from 4.2 to 6.8 per 1,000 births, whereas low-and-middle income countries experience stillbirth ranging from 20 to 32 per 1,000 births [11]. The 2007 Ghana Maternal Health Survey (GMHS) Report [12] indicated that out of the 8,322 pregnancies recorded during the five-year period preceding the survey, more than four in five pregnancies ended in a live birth (82 %). 9 % of the pregnancies were lost through miscarriage, followed by induced abortion (7 %), and stillbirth (2 %) [12, 13]. This situation did not change and was even higher in 2017, where 76 % of pregnancies among women aged 15–49 in the 5 years preceding the survey ended in a live birth, 2 % resulted in a stillbirth, 12 % were miscarried, and 10 % resulted in induced abortion [13].

Various issues surround the recording and classification of induced abortions, miscarriages and stillbirths in most low and middle-income countries, including Ghana [14]. These include limited early evaluation and documentation of risk factors, limited early dating to prevent post-date miscarriages and stillbirths, a limited availability of diagnostic testing, a large proportion of deliveries occurring outside of health facilities, lack of early identification of fetal distress and prompt delivery of such foetuses [15] and incomplete health records.

Despite the wide recognition that stillbirths, induced abortions and miscarriages are common adverse pregnancy outcomes in Ghana [14, 16], it appears empirical data on their risk factors are scanty. The few studies that have been carried out have found socio-economic (level of education, place of residence, wealth status, media exposure), demographic (age, religious beliefs, occupation) and biological (parity) predictors of stillbirths, induced abortions and miscarriages in Ghana [14–22].

Our study seeks to contribute to existing literature on induced abortions and stillbirths and the scanty literature on miscarriage in Ghana by assessing their prevalence and associated factors using the 2017 GMHS. This study would not only present current evidence but would offer empirical basis required to mitigate the burden of adverse maternal health outcomes in Ghana. This study will also identify plausible maternal and newborn interventions that would facilitate Ghana’s prospects of achieving Sustainable Development Goal (SDG) three.

## Methods

### Data extraction

Data for the study was obtained from the second round of the GMHS, which was conducted in 2017. The 2017 GMHS was implemented by the Ghana Statistical Service (GSS) with technical support from Inner City Fund (ICF) through the Demographic and Health Survey (DHS) program. The sampling frame used was from the 2010 Population and Housing Census (PHC) conducted in Ghana. All women aged 15–49 years who were permanent residents of selected households or visitors who stayed in selected households the night before the survey were eligible to participate in the study. The study adopted a multistage stratified cluster sampling. The first stage involved the selection of enumeration areas and households. The detailed description of the survey procedures and the questionnaires used for the data collection can be found in the final report of the survey [13]. A total of 25,062 women participated in the survey. However, by keeping the same sample size for all women with pregnancy history who had complete information on all the variables used in this study, the sample for our study was 18,114. Women with no pregnancy history were excluded from our study. In the GMHS survey, the sample was selected with unequal probability and hence there was reduced sample variability for the subgroups. As a result, adjustment factor, that is weight, was applied to obtain results that were representative [22, 23]. We relied on the “Strengthening the Reporting of Observational Studies in Epidemiology” (STROBE) statement writing the manuscript. The dataset is freely available for download at: https://dhsprogram.com/data/dataset/Ghana_Special_2017.cfm?flag=0.

### Measurement of variables

#### Dependent variables

The dependent variables for the study were induced abortions, miscarriages and stillbirths. They were derived from three questions. The first question was the number of induced abortions since 2012. The responses were 0, 1, 2, 3, 4, 5 and 6. The second was the number of miscarriages since 2012. The responses were 0, 1, 2, 3, 4 and 5. The third question was the number of stillbirths since 2012. The responses were 0, 1, 2, 3, 4 and 5.To get a dichotomous outcome, these three variables were recoded separately as follows; 0 = No and 1 or more = Yes. This categorisation was informed by the literature [2, 6, 8].

#### Independent variables

 Based on the reviewed literature (see [24–26]) and the objective of this study, eleven independent variables were included in the analysis. These were age, educational level, marital status, ethnicity, parity, religion, region, place of residence, exposure to television, exposure to radio and mobile phone use. Age was categorized into, 15–24, 25–34 and 35–49. Educational level was classified into five categories: No formal education = 1, primary school education, Middle/Junior High School (JHS)/ Junior Secondary School (JSS), Senior Secondary School (SSS)/Senior High School (SHS)/Vocational School (VOC) and higher. Marital status was captured as married, cohabiting and never married. Religion was recoded as Christian, Muslim and Other (no religion, Traditionalist). Ethnicity was coded as Akan, Ga/Adangbe, Ewe, Mole–Dagbani and Other. Parity was also captured as 0 birth, 1 birth, 2 births, 3 births and ≥ 4. Residence was coded as urban and rural. Frequency of watching television was captured as not at all, less than once a week and at least once a week. Frequency of listening to radio was categorized as not at all, less than once a week and at least once a week. Mobile phone ownership was captured as yes or no.

### Statistical analyses

The study employed descriptive, bivariate and multivariable data analysis. First of all, descriptive statistics of frequencies and percentages were carried out and the results were used to generate a bar chart that showed the prevalence of induced abortion, miscarriage and stillbirth among women in Ghana. This was followed by a bivariate analysis using Pearson chi–square test to examine the relationship between the socio-demographic characteristics and induced abortion, stillbirth and miscarriage. After this, we carried out a multicollinearity test using Variance Inflation Factor (VIF) and found no evidence of high correlation among the variables. Next, multivariable binary logistic analyses were conducted to assess the risk factors for induced abortion, stillbirth and miscarriage and the results are displayed in Table 2. Only variables that were statistically significant at the bivariate level were included in the models. Model I, II and III show results of the multivariable analysis of the factors associated with induced abortions, miscarriages and stillbirths respectively. The study employed binary logistic regression because this technique permits extrapolation on a combination of continuous and categorical variables. In the bivariate analysis, variables with *p* values < 0.05 were simultaneously included in each of the multivariable logistic regression models. Statistical significance was set at a *p*-value < 0.05. Adjusted odds ratios (AORs), with their 95 % confidence intervals (CIs) were calculated. Analyses were done in Stata/SE 14.0 for Mac (Stata Corp LLC, College Station, Texas USA).

### Ethical consideration

The GMHS 2017 reports that since the study involved participation of human subjects, the ICF Institutional Review Board (IRB) approved the protocol for the survey. The survey indicated that both oral and written informed consent were obtained from the respondents. Nonetheless, since the researchers were not directly involved in the data collection, they sought permission from ICF Macro for the use of the dataset in this study and the terms of use have been strictly observed.

## Results

### Sample characteristics

A total of 25,062 women participated in the 2017 GMHS, with a total of 18,114 women included in this analysis after excluding women with no pregnancy history (6,948). Out of the 18,114 women included in this study, most of them were aged 35–49 (43.8 %), had middle/JHS/JSS education (40.0 %), were married (48.7 %), Christians (79.7 %), Akans (49.2 %) and had three children (29.8 %). The majority of the respondents were from the Ashanti region (19.0 %), listened to radio at least once a week (51.6 %), watched television at least once a week (57.7 %) and owned mobile phone (74.4 %) (see Table 1).

**Table 1:** Sample characteristics and association between socio-demographic characteristics, induced abortion, miscarriage and stillbirth among women in Ghana (*N* = 18,114)

Variables	n (%)	Induced abortion n (%)	*p*-values	Miscarriage n (%)	*p*-values	Stillbirth n (%)	*p*-values
**Age**			< 0.001		< 0.001		0.004
15–24	3190 (17.6)	693 (21.7)		348 (10.9)		76 (2.4)	
25–34	6982 (38.6)	866 (12.4)		905 (13.0)		155 (2.2)	
35–49	7941 (43.8)	318 (4.0)		698 (8.8)		129 (1.6)	
**Educational level**			< 0.001		< 0.001		0.855
No formal education	4341 (24.0)	162 (3.7)		329 (7.5)		77 (1.8)	
Primary	3143 (17.4)	298 (9.5)		309 (9.8)		63 (2.0)	
Middle/JHS/JSS	7239 (40.0)	872 (12.1)		826 (11.4)		151 (2.1)	
SSS/SHS/VOC	2311 (12.8)	410 (17.7)		331 (14.3)		45 (2.0)	
Higher	1080 (6.0)	136 (12.6)		156 (14.4)		23 (2.2)	
**Marital status**			< 0.001		< 0.001		< 0.001
Married	8814 (48.7)	437 (5.0)		981 (11.1)		193 (2.2)	
Cohabiting	4968 (27.4)	681 (13.7)		621 (12.5)		119 (2.4)	
Never married	4331 (23.9)	760 (17.5)		349 (8.1)		48 (1.1)	
**Religion**			< 0.001		< 0.001		0.908
Christianity	14,428 (79.7)	1703 (11.8)		1541 (10.7)		288 (2.1)	
Muslim	2782 (15.4)	127 (4.6)		332 (11.9)		54 (2.0)	
Other	90 (5.0)	48 (5.3)		79 (8.7)		18 (2.0)	
**Ethnicity**			< 0.001		< 0.001		0.400
Akan	8918 (49.2)	1134 (12.7)		975 (10.9)		203 (2.3)	
Ga-Dagme	1365 (7.5)	170 (12.5)		117 (8.6)		16 (1.2)	
Ewe	2497 (13.8)	285 (11.4)		298 (12.0)		38 (1.5)	
Mole-Dagbani	2756 (15.2)	144 (5.2)		299 (10.9)		54 (2.0)	
Others	2579 (14.2)	148 (5.7)				50 (2.0)	
**Parity**			< 0.001		< 0.001		< 0.001
0	1117 (6.2)	533 (47.8)		357 (31.9)		53 (4.8)	
1	3912 (21.6)	506 (12.9)		439 (11.2)		62 (1.6)	
2	3389 (18.7)	355 (10.5)		371 (11.0)		68 (2.0)	
3	5405 (29.8)	338 (6.3)		487 (9.0)		86 (1.6)	
4+	4300 (23.7)	146 (3.4)		298 (6.9)		92 (2.1)	
**Region**			< 0.001		< 0.001		0.030
Western	2366 (13.1)	315 (13.3)		253 (11.1)		48 (2.0)	
Central	1655 (9.1)	156 (9.4)		177 (10.7)		48 (2.9)	
Greater Accra	3119 (17.2)	436 (14.0)		340 (10.9)		41 (1.3)	
Volta	1475 (8.1)	123 (8.4)		145 (9.9)		18 (1.2)	
Eastern	1852 (10.2)	157 (8.5)		187 (10.1)		30 (1.6)	
Ashanti	3423 (19.0)	452 (13.1)		448 (13.0)		95 (2.8)	
Brong Ahafo	1779 (9.8)	186 (10.4)		184 (10.4)		33 (1.8)	
Northern	1362 (7.5)	22 (1.6)		108 (7.9)		30 (2.2)	
Upper east	624 (3.4)	13 (2.1)		53 (8.4)		9 (1.4)	
Upper West	440 (2.4)	18 (4.2)		46 (10.5)		10 (2.3)	
**Residence**			< 0.001		< 0.001		0.522
Urban	9503 (52.5)	1228 (12.9)		1057 (11.1)		176 (1.9)	
Rural	8611 (47.5)	649 (7.5)		894 (10.4)		184 (2.1)	
**Frequency of listening to radio**			< 0.001		< 0.001		0.261
Not at all	4068 (22.5)	349 (8.6)		371 (9.1)		87 (2.1)	
Less than once a week	4700 (26.0)	553 (11.8)		535 (11.4)		82 (1.7)	
At least once a week	9346 (51.6)	975 (10.4)		1045 (11.2)		192 (2.2)	
**Frequency of watching television**			< 0.001		< 0.001		0.136
Not at all	4308 (23.8)	209 (4.9)		370 (8.6)		83 (1.9)	
Less than once a week	3361 (18.6)	292 (8.7)		360 (10.7)		67 (2.0)	
At least once a week	10,446 (57.7)	1377 (13.2)		1222 (11.7)		211 (2.1)	
**Owns mobile phone**			< 0.001		< 0.001		0.810
No	4645 (25.6)	288 (6.2)		368 (7.9)		105 (2.3)	
Yes	13,469 (74.4)	1590 (11.8)		1582 (11.8)		256 (1.9)	

Source: 2017 GMHS dataset

NB: *p*-values generated from Pearson’s chi-square test

### Prevalence of induced abortions, stillbirths and miscarriages in Ghana

**Fig. 1:**
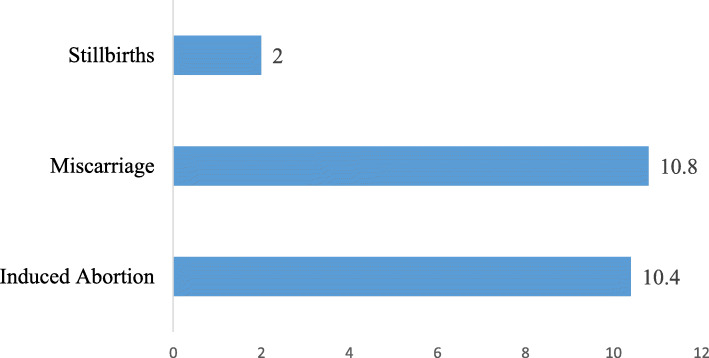
Prevalence of induced abortion, miscarriage and stillbirths among women of reproductive age in Ghana

The prevalence of miscarriages, induced abortions and stillbirth were 10.8 %, 10.4 % and 2 % respectively (see Fig. 1).

### Association between socio-demographic characteristics, induced abortions, miscarriages and stillbirths among women in Ghana

Results of the chi-square test showed that all the socio-demographic variables had significant associations with induced abortions and miscarriages at 95 % confidence interval. However, with stillbirths, only age, marital status, parity and region had significant associations (see Table 1).

### Risk factors for induced abortions, miscarriages and stillbirths among women in Ghana

The results of the multivariable analysis of the risk factors for induced abortions, miscarriages and stillbirths among women in Ghana are presented in Table 2. It was found that women aged 35–49 (AOR = 0.26, 95 % CI = 0.21-32), Muslims (AOR = 0.65, 95 % CI = 0.52–0.82), women with parity 1 or more, those from the Northern region (AOR = 0.30, 95 % CI = 0.21–0.42) and those in urban areas (AOR = 0.76, 95 % CI = 0.66–0.87) were less likely to have induced abortions, compared to those aged 15–19, Christians, those with parity 0 and those who lived in the Ashanti region and in rural areas, respectively. Conversely, women who were cohabiting (AOR = 2.02, 95 % CI = 1.70–2.39) and those who watched television at least once a week (AOR = 1.57, 95 % CI = 1.30–1.89) were more likely to have induced abortion, compared to those who were married and those who do not watch television at all. With miscarriage, the highest odds were found among women aged 25–34 (AOR = 1.62, 95 % CI = 1.39–1.89), those with higher level of education (AOR = 1.42, 95 % CI = 1.13–1.78), Muslims (AOR = 1.31, 95 % CI = 1.13–1.52), those of the Mole-Dagbani ethnic group (AOR = 1.27, 95 % CI = 1.05–1.54), rural dwellers (AOR = 1.14, 95 % CI = 1.01–1.29) and those who watched television less than once a week (AOR = 1.20, 95 % CI = 1.02–1.41), compared to those aged 15–19, those with no formal education, Christians, Akans, urban dwellers and those who never watched television. Women who had never married (AOR = 0.34, 95 % CI = 0.23–0.51) and those who had one or more parity were less likely to have stillbirths, compared to those who were married and those with parity zero. Marital status and parity were the common risk factors for induced abortions, miscarriages and stillbirths.

**Table 2:** Multivariable analysis of the risk factors for induced abortion, miscarriage and stillbirth among women in Ghana

Explanatory variables	Model I Induced abortion Adjusted Odds Ratio (AOR): 95 % CI	Model II Miscarriage Adjusted Odds Ratio (AOR): 95 % CI	Model III Stillbirth Adjusted Odds Ratio (AOR): 95 % CI
**Age**			
15–24	Ref	Ref	Ref
25–34	0.65^***^ [0.56–0.76]	1.62^***^ [1.39–1.89]	0.97 [0.71–1.34]
35–49	0.26^***^[0.21–0.32]	1.53^***^[1.27–1.84]	0.74 [0.51–1.07]
**Educational level**			
No education	Ref	Ref	-
Primary	1.22 [0.97–1.54]	1.28^**^[1.08–1.51]	-
Middle/JHS/JSS	1.18 [0.95–1.47]	1.32^***^[1.13–1.55]	-
SSS/SHS/VOC	1.25[0.97–1.60]	1.30^**^[1.07–1.58]	-
Higher	1.06 [0.77–1.45]	1.42^**^[1.13–1.78]	-
**Marital status**			
Married	Ref	Ref	Ref
Cohabiting	2.02^***^[1.70–2.39]	0.96 [0.84–1.10]	0.84 [0.63–1.13]
Never married	1.99^***^[1.69–2.35]	0.42^***^[0.35–0.49]	0.34^***^[0.23–0.51]
**Religion**			
Christianity	Ref	Ref	-
Muslim	0.65^***^[0.52–0.82]	1.31^***^[1.13–1.52]	-
Other	0.74[0.51–1.08]	1.08 [0.84–1.39]	-
**Ethnicity**			
Akan	Ref	Ref	
Ga-Dagme	0.97 [0.75–1.28]	0.93 [0.71–1.22]	-
Ewe	1.03 [0.81–1.30]	1.23 [1.00-1.52]	-
Mole-Dagbani	0.90 [0.70–1.16]	1.27^*^[1.05–1.54]	-
Others	0.89 [0.71–1.12]	1.12 [0.93–1.36]	-
**Parity**			
0	Ref	Ref	Ref
1	0.14^***^[0.11–0.17]	0.20^***^[0.17–0.24]	0.27^***^[0.18–0.40]
2	0.14^***^[0.11–0.17]	0.17^***^[0.14–0.20]	0.25^***^[0.17–0.38]
3	0.13^***^[0.11–0.16]	0.13^***^[0.10–0.15]	0.22^***^[0.15–0.32]
4+	0.14^***^[0.10–0.18]	0.09^***^[0.07–0.12]	0.26^***^[0.17–0.40]
**Region**			
Western	Ref	Ref	Ref
Central	0.66^**^[0.51–0.86]	1.03 [0.80–1.32]	1.47 [0.89–2.40]
Greater Accra	0.91 [0.72–1.17]	0.88 [0.69–1.11]	0.80 [0.47–1.35]
Volta	0.78 [0.56–1.08]	0.87 [0.65–1.17]	0.71 [0.37–1.33]
Eastern	0.63^***^[0.49–0.82]	0.92 [0.72–1.17]	0.85 [0.50–1.45]
Ashanti	0.91 [0.73–1.13]	1.14 [0.92–1.39]	1.34 [0.86–2.08]
Brong Ahafo	0.78^*^ [0.62–0.99]	0.98 [0.78–1.23]	0.86 [0.52–1.43]
Northern	0.30^***^[0.21–0.42]	0.70^***^[0.55–0.89]	1.00 [0.64–1.53]
Upper East	0.32^***^[0.22–0.46]	0.69^**^[0.53–0.90]	0.65 [0.38–1.09]
Upper West	0.62^**^[0.44–0.86]	0.95 [0.74–1.22]	1.09 [0.68–1.74]
**Residence**			
Urban	Ref	Ref	-
Rural	0.76^***^[0.66–0.87]	1.14^*^[1.01–1.29]	-
**Frequency of listening to radio**			
Not at all	Ref	Ref	-
Less than once a week	1.06 [0.89–1.27]	1.07 [0.92–1.25]	-
At least once a week	0.93 [0.79–1.10]	1.06 [0.93–1.21]	-
**Frequency of watching television**			
Not at all	Ref	Ref	-
Less than once a week	1.13 [0.91–1.41]	1.20^*^[1.02–1.41]	-
At least once a week	1.57^***^[1.30–1.89]	1.12 [0.97–1.29]	-
**Owns mobile phone**			
No	Ref	Ref	-
Yes	1.42^***^[1.20–1.68]	1.22^***^[1.07–1.40]	-

Source: 2017 GMHS dataset. CI = Confidence, Interval in square brackets; Ref = reference; * *p* < 0.05, ** *p* < 0.01, *** *p* < 0.001

NB: Columns with dash (-) shows that those variables were not included in the regression model since they were not significant in the chi-square test

## Discussion

This study sought to examine the factors associated with induced abortions, miscarriages and stillbirths in Ghana. We found age, educational level, marital status, religion, parity, region, place of residence and exposure to media as factors associated with both induced abortion and miscarriage whiles marital status and parity were identified as factors associated with stillbirths. Importantly, marital status and parity were found as key factors associated with all the three pregnancy outcomes.

In terms of induced abortion, we found age, marital status, religion, place of residence and parity as the major associated factors. Specifically, induced abortion was found to be high among young women, never married and cohabiting women, Christians, women with parity zero and those who lived in urban areas. Previous studies in Ghana have identified similar socio-demographic factors associated with induced abortion [21, 22, 27]. In terms of the association between age, marital status and induced abortion, young women aged 15–24 in Ghana are likely to be in school or might be pursuing the compulsory national service. These persons are mostly not married and dependent on their parents and unlikely to desire children. They may therefore resort to induced abortion in the event of pregnancy in order not to add to the economic burden of their families. Also, the fact that child marriage (under 18) is unacceptable in Ghana [28] and societies take no pleasure in childbearing before wedlock in Ghana [29] could further dissuade them from giving birth and hence terminate any pregnancy that occur at this age. Hence, induced abortion is more likely to happen among young women who are never married or cohabiting. A possible explanation for the finding that induced abortion is high among women with parity zero could be that these women might have terminated their previous pregnancies, the reason for their zero parity. Again, women with parity zero may consider themselves as not ready to give birth and as a result may opt for an induced abortion to be able to prepare themselves adequately before giving birth [21]. Our finding that Christians are more likely to have induced abortions, compared to Muslims needs further exploration through qualitative research since both religious groups do not support induced abortion.

With miscarriage, we identified key socio-economic (level of education and place of residence) and demographic (age, marital status, parity and religion) associated factors. For the socio-economic factors, we found a high prevalence of miscarriages among women in rural areas and those with at least primary education. In rural areas, miscarriages have been reported by some studies [26, 30] and a major contributory factor has been identified as poor sanitation conditions in rural settings [26]. On the association between level of education and miscarriage, it is common knowledge that education offers several benefits to maternal health and encompassing provision of knowledge about fertility regulation, best maternal health practices and quality newborn care [31]. Yet, highly educated women may be in managerial positions with heavy workloads. Consequently, they may not get quality time to monitor their diet and adhere to the recommended pregnancy precautions such as being consistent at antenatal care among others. Evidence indicate that striving a balance between child bearing and work demands has been the bane of a number of highly educated women [32]. In terms of age, marital status and miscarriage, miscarriage has been considered as a natural event and women who experience miscarriage might have been exposed to risk factors that they may even not be aware of. Such risk factors often include stress from their partners, families or struggling to meet routine deadlines at work as established in the literature [33–35] and these occur among older women who are also more likely to be married. The association between high parity and reduced likelihood of miscarriage has been reported by Shaimaa and Shukriya [36]. The authors explained that women with higher parity may have the childbearing experience to take care of their pregnancies and reduce the chances of miscarriage compared to those with parity zero [36]. Further research is needed to unearth the reason for the high prevalence of miscarriage among Muslim women compared to Christians.

We found marital status and parity as the key factors associated with stillbirths. Our finding that unmarried women are less likely to experience stillbirths compared to married women contradict the findings of previous studies that identified higher risk of stillbirths among unmarried women compared to married women [37, 38]. We argue that just like miscarriages, stillbirths are also natural events and women who experience stillbirth may have been exposed to risk factors such as stress from their partners, families or struggling to meet routine deadlines at work as established in the literature [33–35] and these stressful events are more likely to occur in marriages. In relation to our finding that the odds of stillbirth is lower among women with higher parity, it contradicts the findings of studies that have identified that high chances of stillbirth characterise women with multiple pregnancies [39–41] and those that have found a u-shaped relationship between parity and stillbirths [42, 43]. Notwithstanding, the possible reason for our finding could be explained in terms of pregnancy experience obtained by women with higher parity which could empower them to observe essential health practices that protect them from stillbirths.

### Strengths and limitations

We acknowledge that there are some methodological limitations in our study. The cross-sectional nature of the study limits causal inference and also it is possible that the miscarriages and stillbirths as reported could be underreported or over reported possibly due to recall bias or hesitancy in disclosure. Again, this study could only examine the socio-demographic factors associated with induced abortion, miscarriage and stillbirth as the dataset did not have other variables such as maternal hypertensive disorder, diabetes, infections and fetal growth restriction which have been found as risk factors for adverse pregnancy outcomes and induced abortions. In spite of these, the sound sampling approach and the nationally representative nature of the study offers a much reliable reflection of the current situation of induced abortions, miscarriages and stillbirths in Ghana.

## Conclusions

Our study indicates that efforts to mitigate induced abortions, miscarriages and stillbirths in Ghana need to focus on women in unions, women between 30 and 39 years, those with mobile phones as well as rural dwellers. Ability of the nation to reconsider its generic maternal health interventions to target the identified category of women would have much positive prospects for the country. Similarly, other sub-Saharan African countries experiencing induced abortions, miscarriages and stillbirths can consider these categories of women and streamline targeted interventions utilising television and other mass media avenues to reach such women. Synergy between government health institutions and the private sector cannot be left out if much success can be achieved in efforts to subside the current prevalence of induced abortions, stillbirths and miscarriages confronting the country. Therefore, efforts by the government to limit induced abortions, miscarriages and stillbirths in Ghana need to focus on the disparities in socio-demographic characteristics of women.

## Data Availability

The dataset is freely available for download at: https://dhsprogram.com/data/dataset/Ghana_Special_2017.cfm?flag=0.

## Abbreviations

AOR: Adjusted Odds Ratio
CI: Confidence Interval
DHS: Demographic and Health Survey
GMHS: Ghana Maternal Health Survey
ICF: Inner City Fund
PHC: Population and Housing Census
STROBE: Strengthening the Reporting of Observational Studies in Epidemiology
